# The role of patch testing in non-immediate drug hypersensitivity reactions

**DOI:** 10.1186/1939-4551-8-S1-A2

**Published:** 2015-04-08

**Authors:** Razvigor Darlenski, Nikolai Tsankov

**Affiliations:** 1 Tokuda Hospital, Sofia, Bulgaria

## Background

Skin is the most commonly affected organ by adverse drug reactions in almost 30% of all cases. According to the international consensus on drug allergy, drug hypersensitivity reactions (DHRs) constitute 15% of all adverse drug reactions affecting more than 7% of the general population.

## Methods

Herein we present case series of various non-immediate DHRs (occurring at any time as from 1 hour after the initial drug administration) amongst which drug-related intertriginous and flexural exanthema (Baboon syndrome), DRESS syndrome, fixed drug eruption and contact dermatitis. In all cases we performed patch testing with standardized and allergens prepared at our laboratory with the suspected drugs. The timing of patch test procedure was in median 6 weeks after the resolution of skin changes.

## Results

Based on the patch test results, we comment on the sensitivity and specificity of patch test in non-immediate DHRs. Different variables influence patch testing in DHRs such as allergen preparation, vehicle, concentration, intake of certain medications and testing healthy controls with the suspected allergens. We emphasize on these and on the demand for control and interpretation of the results. The role of web drug allergen databases in preparation of proper drug concentration and vehicle for patch testing is reviewed.

## Conclusions

This presentation reports the personal experience with in vivo skin patch testing in patients with non-immediate DHRs.

